# Measuring refractive index distribution in the human eye lens

**DOI:** 10.18632/oncotarget.6322

**Published:** 2015-11-13

**Authors:** James M. Pope, David A. Atchison

**Affiliations:** School of Chemistry, Physics and Mechanical Engineering, Faculty of Science and Engineering, Queensland University of Technology, Brisbane, Queensland, Australia

**Keywords:** eye lens, refractive index, accommodation, MRI, X-ray Talbot Interferometry, Gerotarget

In a recent article, Pierscionek et al. [1] reported non-invasive measurements of the age dependence of refractive index distribution in human eye lenses *in vitro* using a novel X-ray Talbot Interferometry method. In their paper, the authors make frequent reference to our own work in which we employed magnetic resonance imaging (MRI) to make similar non-invasive measurements of the refractive index distribution in the human eye lens [2, 3]. Prior to the current work, ours was the only method for making such measurements both non-invasively and without prior assumptions about the shape of the refractive index distribution. For this reason, the latest work is to be welcomed. However at several points in the paper, Pierscionek et al. [1] make statements about our technique which are factually incorrect. Specifically, they state that the MRI technique is sensitive only to free water and does not account for bound water, the proportion of which may vary with age in the eye lens. In fact, measurements of the transverse relaxation time T2 on which our method is based, are sensitive to a weighted average of the properties of both bound and free water protons, because on the timescale of the measurements (typically tens of milliseconds) there is rapid exchange of the water molecules between bound and free environments. Furthermore, T2 values in the eye lens are dominated by proton exchange between the bound water molecules and amide or hydroxyl groups on the crystallin proteins. For this reason there is a correlation between the measured transverse relaxation rate (R2 = 1/T2) and the concentration of crystallins, which determines the refractive index of the lens. The correlation between transverse relaxation rate (R2 = 1/T2) and protein concentration is characteristic of protein solutions [4]. Most importantly, the criticisms by Pierscionek et al. of our method are irrelevant because our technique for measuring refractive index depends only on an empirical relationship between R2 and refractive index, which we determined from human eye lens homogenates covering a range of concentrations, using spectroscopic NMR measurements of T2 and corresponding refractive index data obtained with an Abbe refractometer [3].

The results of Pierscionek et al. [1] with respect to refractive index distribution are broadly in agreement with our earlier study [3]. It should be emphasised that a significant problem in interpreting data from human eye lenses *in vitro* arises from the fact that the state of accommodation of the lenses is uncertain and varies with age of the lens. For young lenses in particular, the lenses are likely to be in a fully accommodated state due to the elastic properties of the lens capsule, but they may in fact exhibit higher accommodation than is possible *in vivo* if in the latter case there remains residual tension in the zonules that support the lens and connect it to the ciliary muscles. In order to measure both the refractive index distribution through the lens at a known state of accommodation and changes in the distribution with degree of accommodation, it is therefore essential to make measurements *in vivo*. Such measurements can be made using the MRI technique [5, 6], but are not practicable with a method that employs X-ray (synchrotron) radiation. In vivo measurements may also be essential if the goal of personalised lens implants is to be realised in future.
